# Streptozotocin-Induced Type 1 and 2 Diabetes Mellitus Mouse Models Show Different Functional, Cellular and Molecular Patterns of Diabetic Cardiomyopathy

**DOI:** 10.3390/ijms24021132

**Published:** 2023-01-06

**Authors:** Fabiola Marino, Nadia Salerno, Mariangela Scalise, Luca Salerno, Annalaura Torella, Claudia Molinaro, Antonio Chiefalo, Andrea Filardo, Chiara Siracusa, Giuseppe Panuccio, Carlo Ferravante, Giorgio Giurato, Francesca Rizzo, Michele Torella, Maria Donniacuo, Antonella De Angelis, Giuseppe Viglietto, Konrad Urbanek, Alessandro Weisz, Daniele Torella, Eleonora Cianflone

**Affiliations:** 1 Department of Experimental and Clinical Medicine, Magna Graecia University, 88100 Catanzaro, Italy; 2 Department of Medical and Surgical Sciences, Magna Graecia University, 88100 Catanzaro, Italy; 3 Department of Precision Medicine, University of Campania “Luigi Vanvitelli”, 80138 Naples, Italy; 4 Department of Medicine, Surgery and Dentistry ‘Scuola Medica Salernitana′, University of Salerno, 84081 Salerno, Italy; 5 Department of Translational Medical Science, University of Campania “L. Vanvitelli”, 80138 Naples, Italy; 6 Department of Experimental Medicine, University of Campania “L. Vanvitelli”, 80138 Naples, Italy; 7 Department of Molecular Medicine and Medical Biotechnology, Federico II University, 88121 Naples, Italy

**Keywords:** type 1 diabetes mellitus, type 2 diabetes mellitus, cardiac cell senescence, streptozotocin, left ventricular remodeling, myocardial regeneration

## Abstract

The main cause of morbidity and mortality in diabetes mellitus (DM) is cardiovascular complications. Diabetic cardiomyopathy (DCM) remains incompletely understood. Animal models have been crucial in exploring DCM pathophysiology while identifying potential therapeutic targets. Streptozotocin (STZ) has been widely used to produce experimental models of both type 1 and type 2 DM (T1DM and T2DM). Here, we compared these two models for their effects on cardiac structure, function and transcriptome. Different doses of STZ and diet chows were used to generate T1DM and T2DM in C57BL/6J mice. Normal euglycemic and nonobese sex- and age-matched mice served as controls (CTRL). Immunohistochemistry, RT-PCR and RNA-seq were employed to compare hearts from the three animal groups. STZ-induced T1DM and T2DM affected left ventricular function and myocardial performance differently. T1DM displayed exaggerated apoptotic cardiomyocyte (CM) death and reactive hypertrophy and fibrosis, along with increased cardiac oxidative stress, CM DNA damage and senescence, when compared to T2DM in mice. T1DM and T2DM affected the whole cardiac transcriptome differently. In conclusion, the STZ-induced T1DM and T2DM mouse models showed significant differences in cardiac remodeling, function and the whole transcriptome. These differences could be of key relevance when choosing an animal model to study specific features of DCM.

## 1. Introduction

The prevalence of diabetes mellitus (DM) is continuously increasing at a frightening rate. The World Health Organization (WHO) reported that 108 million adults affected by DM in 1980 increased to 422 million in 2014, and this trend is only steeply increasing [1,2]. The main cause of morbidity and mortality in diabetic patients is cardiovascular complications [3,4,5]. DM increases the risk of heart failure (HF) up to fivefold [6,7,8,9,10]. The clinical outcomes associated with HF are considerably worse for patients with DM than for those without. However, the high prevalence of HF in diabetic patients is not explained just through other cardiac risk factors, such as atherosclerotic coronary artery disease, hypertension and valvular disease. Indeed, in the absence of such risk factors, individuals with DM can show abnormal myocardial structure and performance, defined as diabetic cardiomyopathy (DCM) [11,12]. In its early stages, DCM starts with subclinical structural and functional abnormalities, including left ventricular (LV) hypertrophy, fibrosis and cell-signaling alterations. These pathophysiological changes of cardiac fibrosis and stiffness and associated subclinical diastolic dysfunction evolve into HF with normal ejection fraction and, eventually, systolic dysfunction accompanied by HF with reduced ejection fraction [11,13,14]. The mechanisms that underlie development of DCM are, however, multifactorial and incompletely understood.

DM has adverse effects on the different cell types of the heart, including cardiomyocytes. Various small- and large-animal models of type 1 and type 2 DM (T1DM and T2DM, respectively) have been created to evaluate the effects of diabetes on the heart. These models were established through genetic manipulations, dietary interventions and treatment with pancreatic toxins, all of which mimic several aspects of DM and DCM. Streptozotocin (STZ) is a glucosamine–nitrosourea antibiotic that is toxic to pancreatic β-cells [15,16,17]. Following intraperitoneal injection, STZ, based on its structural similarity to glucose, is carried into pancreatic β-cells via glucose transporter 2 (GLUT2), resulting in β-cell necrosis and ensuing total or partial loss of insulin production, depending on the STZ dose and the animal age at STZ administration [18,19]. STZ models are used to study both T1DM and T2DM, whereby high-dose STZ protocols are primarily used to study T1DM, as the latter STZ regime creates severe and extensive β-cell necrosis with practical total loss of pancreatic insulin secretion [17]. Owing to the low efficiency of T2DM development with only high-fat-diet (HFD) chow feeding, more recent models have reproduced clinical presentation of late-stage T2DM, characterized with insufficient insulin production through partial β-cell destruction via addition of a very low dose of STZ [15,17,20].

Despite the specific limitations of the models generated, these STZ-induced DM mouse models mimic various perturbations observed in the diabetic myocardium in fatty acid oxidation, glucose oxidation, mitochondrial content and function, Ca2+ handling, oxidative stress, lipotoxicity, RAAS activation, inflammation, AGE, ER Stress, autophagy, cell death, fibrosis and contractile function and size [20,21]. Therefore, these DM mouse models continue to provide important mechanistic insight into the pathogenesis that underlies DCM. Nevertheless, a head-to-head comparison of these two widely used DM mouse models for their effects on cardiac remodeling and function could be key to better choose the more suitable among the two when addressing specific hypotheses and questions related to pathogenesis and treatment of DMC. Therefore, this study addressed this gap of direct evidence.

## 2. Results

### 2.1. STZ-Based T1DM and T2DM Mouse Models Affected Global Left Ventricular Function Differently

To assess whether STZ-derived mouse models of type 1 and type 2 DM cause different phenotypes of cardiomyopathy, a single high dose of STZ (200 mg/Kg) was injected into 10-week-old male and female C57BL/6J mice to cause a total depletion of pancreatic β cells, generating T1DM mice [16]. T2DM mice were generated through HFD for four weeks, starting at six weeks of age, and, during the fourth week, via daily injections of low-dose STZ (40 mg/Kg) over four consecutive days [22]. Control mice were fed with a normal chow diet (NCD) or an HFD without STZ. Four weeks after high/low doses of STZ, all treated animals were diabetic, showing altered fasting glycemia levels when compared to control mice that were fed with normal as well as high-fat diets (NCD = 150.1 ± 18.9 mg/dL; HFD = 155.5 ± 14.06 mg/dL; T1DM = 455.3 ± 69.3 mg/dL; T2DM = 345.8 ± 70.2 mg/dL; *p*-value < 0.0001). Hyperglycemia was maintained through eight weeks after STZ administration in both T1DM and T2DM (T1DM = 440.6 ± 57.3 mg/dL; T2DM = 351 ± 63.8 mg/dL). 

To assess whether STZ-based T1DM and T2DM affected cardiac systolic and diastolic function differently or similarly, in vivo M-mode parasternal long-axis echocardiography and flow and tissue Doppler imaging were performed at eight weeks following STZ treatment. Considering that HFD per se does not alter histology or cardiac structure or function over twelve weeks (see [22]), to simplify presentation of these data, we included in this analysis only mice fed with the normal chow diet as a control (CTRL).

**Figure 1:**
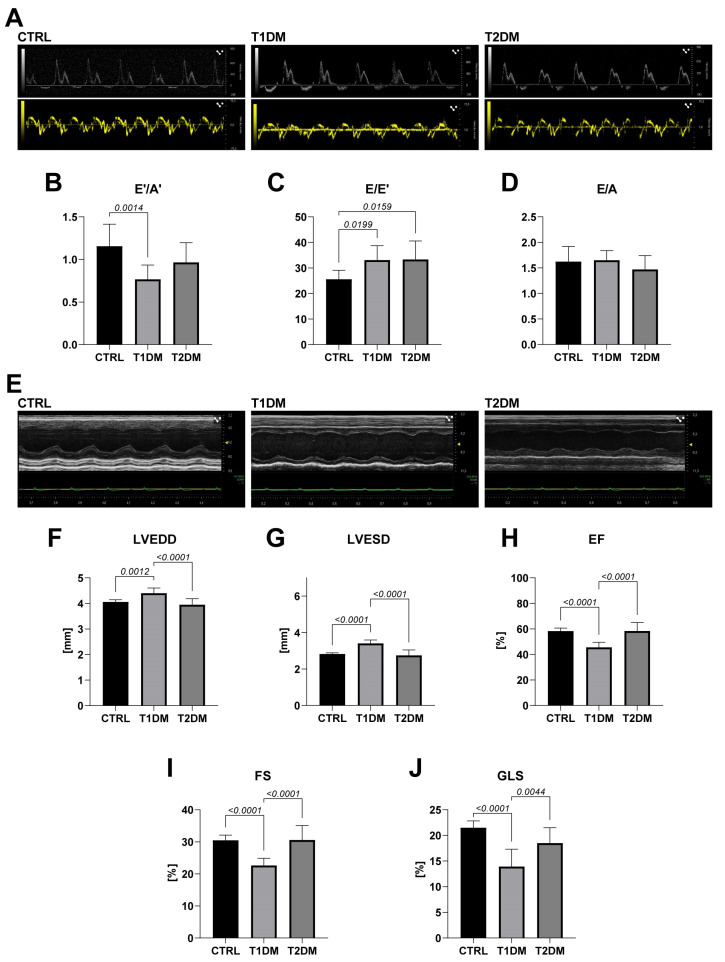
STZ-based T1DM and T2DM mouse models affected left ventricular diastolic and systolic function differently. (**A**) Representative pulsed-wave (PW) Doppler mitral velocity (MV) tracing and PW tissue Doppler imaging (TDI) velocity tracing in T1DM, T2DM and control (CTRL) mice. (**B**–**D**) Cumulative data of diastolic function: E′/A′ ratio (**B**), E/E′ ratio (**C**) and E/A ratio (**D**) in T1DM vs. T2DM vs. CTRL mice (CTRL, n = 10; T1DM, n = 11; T2DM, n = 12). **B**: T1DM vs. CTRL, *p* = 0.0014. **C**: T1DM vs. CTRL, *p* = 0.0199; T2DM vs. CTRL; *p* = 0.0159 (one-way ANOVA with Tukey multiple comparison test). (**E**) Representative M-mode tracing of long-axis left ventricle in in T1DM, T2DM and CTRL mice. (**F**–**J**) Cumulative data of cardiac dimensions and systolic function in T1DM and T2DM when compared to control mice 8 weeks after STZ injection (CTRL, n = 10; T1DM, n = 11; T2DM, n = 12). (**F**) LVEDD = left ventricle and diastolic diameter; (**G**) LVESD = left ventricular end-systolic diameter; (**H**) EF = ejection fraction; (**I**) FS = fractional shortening; (**J**) GLS = global longitudinal strain. **F**: T1DM vs. CTRL, *p* = 0.0012; T1DM vs. T2DM, *p* < 0.0001. **G**: T1DM vs. CTRL, *p* <0.0001; T2DM vs. CTRL, *p* < 0.0001. **H**: T1DM vs. CTRL, *p* <0.0001; T2DM vs. CTRL, *p* < 0.0001. **I**: T1DM vs. CTRL, *p* < 0.0001. **J**: T1DM vs. CTRL, *p* < 0.0001; T1DM vs. T2DM, *p* = 0.0044 (one-way ANOVA with Tukey multiple comparison test). Data are mean ± SD.

As previously reported [22], in the mice with T2DM, an increase in the ratio of early transmitral flow velocity (E wave) to early mitral annulus tissue velocity (E′ wave), an index of left ventricular (LV) filling pressure, was detected (30% increase as compared to CTRL; *p* = 0.0159; Figure 1C and Table 1). This was also evident in the T1DM mice, where a significant reduction in E′ velocity and in the ratio of early to late diastolic mitral annulus tissue velocity was observed (E′/A′; T1DM: 33% decrease as compared to CTRL; *p* = 0.014) (Figure 1B and Table 1). In T1DM vs. T2DM, this reduction was accompanied by a similar increase of the E/E′ ratio (Figure 1C and Table 1), which represents a more reliable and reproducible index of diastolic dysfunction in animal models of cardiomyopathy. Changes in the mitral-valve early- to late-filling-velocity E/A ratio did not reach significance (Figure 1D and Table 1). Representative traces of the mitral-flow Doppler and tissue Doppler velocities are shown in Figure 1A. These data suggest that diastolic dysfunction is a common feature of both STZ-induced T1DM and T2DM cardiomyopathy and is more severe in STZ-induced T1DM mouse models.

Systolic function was assessed through M-mode parasternal long-axis echocardiography. Despite T2DM mice developing diastolic dysfunction, this echocardiography showed that there was no significant difference in the interventricular septum or posterior wall thickness, the LV end-diastolic diameter (LVEDD) or the LV end-systolic diameter (LVESD) in the STZ-treated mice when compared to the CTRL mice (*p* > 0.05) (Figure 1E–G and Table 1). Furthermore, in the T2DM mice, systolic function was preserved, as demonstrated from the normal values of ejection fraction and fractional shortening at eight weeks (Figure 1H,I and Table 1). Overall, these data suggest that an STZ-induced T2DM mouse model leads to development of a model of HF with preserved ejection fraction. 

On the contrary, the STZ-induced T1DM mouse model resulted in an overall worse cardiac remodeling, significantly increasing both the LVEDD and LVESD at 8 weeks, when compared to the CTRL mice (8% and 21% increases, respectively; Figure 1F,G and Table 1). The latter turned into a significant diabetes-induced reduction in ejection fraction (22% decrease) and fractional shortening (26% decrease) in the T1DM mice at eight weeks (Figure 1H,I and Table 1). Therefore, these data suggest that an STZ-induced T1DM mouse model leads to the development of an animal model of HF with reduced ejection fraction.

### 2.2. STZ-Based T1DM and T2DM Mouse Models Affected Myocardial Performance Differently

Although rodent systolic cardiac function has been classically estimated through measuring of ejection fraction and fractional shortening from 2D echocardiography, the recent introduction of speckle-tracking analysis allows for a more sensitive evaluation of cardiac dysfunction [23]. Consistently with the ejection-fraction and fractional-shortening data, in the T1DM mice, global longitudinal strain was depressed 8 weeks after injection of STZ when compared to the CTRL mice (35% reduction; *p* < 0.0001; Figure 1J). On the other hand, in line with the standard echocardiographic evaluation that showed no significant change in EF or FS, speckle-tracking-based strain analysis on the long- and short-axis B-mode demonstrated that STZ-induced T2DM cardiomyopathy did not reduce myocardial contractility, as indeed, global longitudinal values were unaltered at 8 weeks (Figure 1J). Overall, these data suggest that STZ-induced T1DM but not T2DM cardiomyopathy significantly and consistently reduces myocardial performance in mice.

### 2.3. STZ-Based T1DM and T2DM Mouse Models Affected Left Ventricular Remodeling Differently

**Figure 2:**
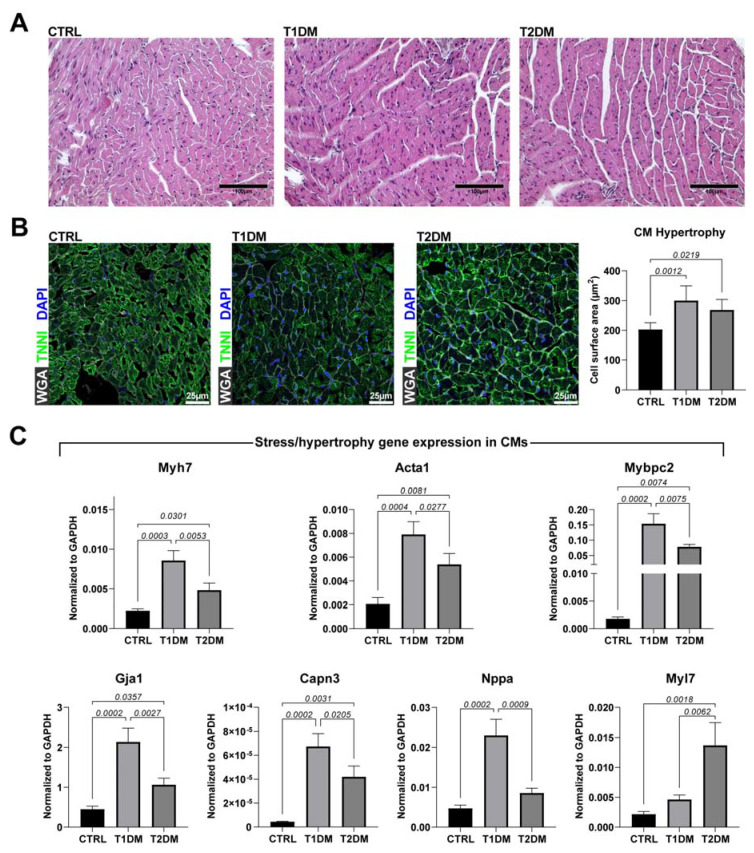
STZ-based T1DM and T2DM mouse models displayed pathological left ventricular remodeling accompanied by stress-induced gene expression and hypertrophy. (**A**) Representative cross-section images of cardiac tissues, from CTRL, T1DM and T2DM mice, stained with hematoxylin and eosin. Scale bar = 100 µm. (CTRL, n = 7; T1DM, n = 8; T2DM, n = 9.) (**B**) Representative confocal images of cardiac cross-sections and bar graph showing cardiomyocyte hypertrophy in T1DM and T2DM mice when compared to CTRL mice (WGA, wheat germ agglutinin, Cy5 staining; cTnI, green; DAPI, blue nuclei). Scale bar = 25 µm. (CTRL, n = 7; T1DM, n = 8; T2DM, n = 9.) (**C**) Bar graphs showing the expressions of stress/hypertrophy genes in cardiomyocytes isolated from CTRL, T1DM and T2DM mice (n = 3). Data are mean ± SD.

STZ-induced DM in animals represents a clinically relevant model to study the pathogeneses of diabetic-derived cardiomyopathy and associated complications [15]. Although the STZ-induced T1DM and T2DM mouse models shared common characteristics, considering that the two models affected cardiac function differently over an 8-week follow-up, we hypothesized that they also affected pathologic cardiac remodeling, defined based on cardiomyocyte hypertrophy, apoptosis and interstitial fibrosis, differently [24]. When compared to CTRL mice, heart sections from the T1DM and T2DM mice showed increased ventricular cardiomyocyte (CM) size (Figure 2A,B). Interestingly, the T1DM mice showed a higher CM area (hypertrophy) when compared to the T2DM counterpart (Figure 2A,B). The increase in CM size in the T1DM versus T2DM mice was further investigated through RT-PCR on freshly isolated CMs from n = 3 additional mice per group (CTRL, T1DM and T2DM mice). Expression levels of stress/hypertrophy-associated genes, such as Myh7, Acta1, Mybpc2, Gja1, Capn3, Nppa and Myl7, in adult CMs from the T1DM and T2DM mice revealed a significant increase when compared to those of adult CMs from the CTRL mice (Figure 2C). Moreover, between the two diabetic groups, we found higher expression levels of Myh7, Mybpc2 and Gja1 in CMs from the T1DM mice when compared to those from the T2DM mice (Figure 2C). On the other hand, Myl7 was upregulated in the T2DM versus T1DM mice (Figure 2C).

**Figure 3:**
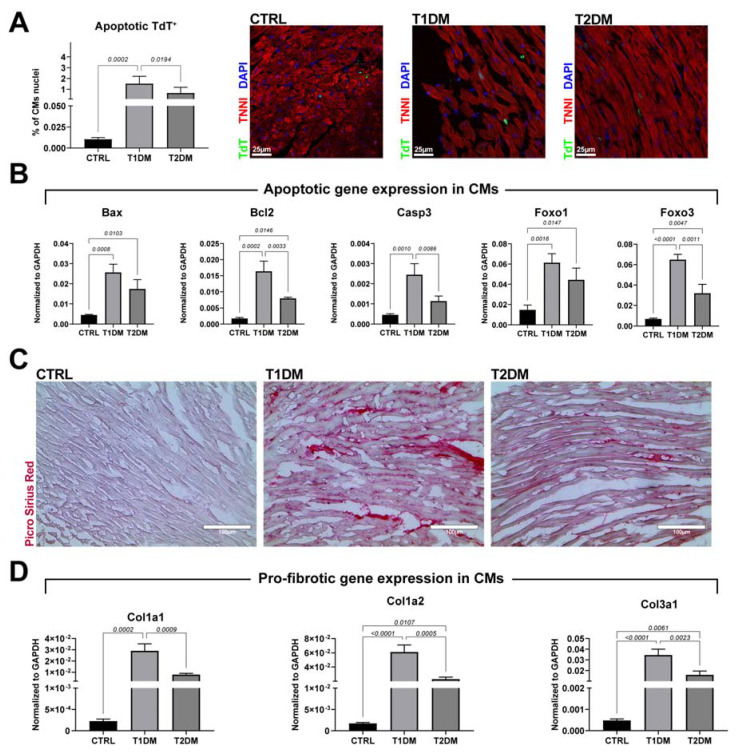
STZ-based T1DM and T2DM mouse models differed in accumulation of reactive interstitial fibrosis in the left ventricular myocardium. (**A**) Bar graph and representative confocal images of apoptotic TdT (green)-positive cardiomyocyte nuclei in T1DM and T2DM mice compared to CTRL mice. Scale bar = 25 µm. (CTRL, n = 7; T1DM, n = 8; T2DM, n = 9.) (**B**) Bar graphs showing the expressions of apoptotic genes in cardiomyocytes isolated from CTRL, T1DM and T2DM mice (n = 3). (**C**) Representative light microscopy of Picrosirius red staining of T1DM and T2DM mice compared to CTRL mice. Scale bar = 100 µm. (CTRL, n = 7; T1DM, n = 8; T2DM, n = 9.) (**D**) Bar graphs showing the expressions of profibrotic genes in cardiomyocytes isolated from CTRL, T1DM and T2DM mice (n = 3). Data are mean ± SD.

Pathological CM hypertrophy was associated with enhanced levels of cell death and myocardial interstitial fibrosis as consequences (Figure 3). An increase in CM death is commonly detected in the early stages of STZ-induced DM in mice [25,26]. Apoptotic DNA fragmentation, evaluated through a TUNEL-based assay, showed an increased percentage of TUNEL-positive CM nuclei in the T1DM versus T2DM mice: 1.5 ± 0.6% vs. 0.6 ± 0.5%, respectively, compared with 0.01 ± 0.01% positive CM nuclei in the CTRL mice (Figure 3A). This finding was further assessed through RT-PCR on freshly isolated CMs from the T1DM, T2DM and CTRL mice, where the expression levels of the apoptotic gene markers Bax, Casp3, Bcl2, Foxo3 and Foxo1 were higher in the T1DM versus T2DM mice and in the T1DM and T2DM mice versus the CTRL mice (Figure 3B).

Pathological CM hypertrophy and cell death were accompanied by enhanced levels of myocardial interstitial fibrosis, assessed with Picrosirius red staining (Figure 3C). The comparison between the T1DM and T2DM mice revealed an increase in myocardial fibrosis in the first group of animals, as confirmed with RT-PCR on freshly isolated CMs, in which the expressions of Col1a1, Col1a2 and Col3a1 were found to be higher in the T1DM mice compared to in the T2DM mice (Figure 3D).

**Figure 4:**
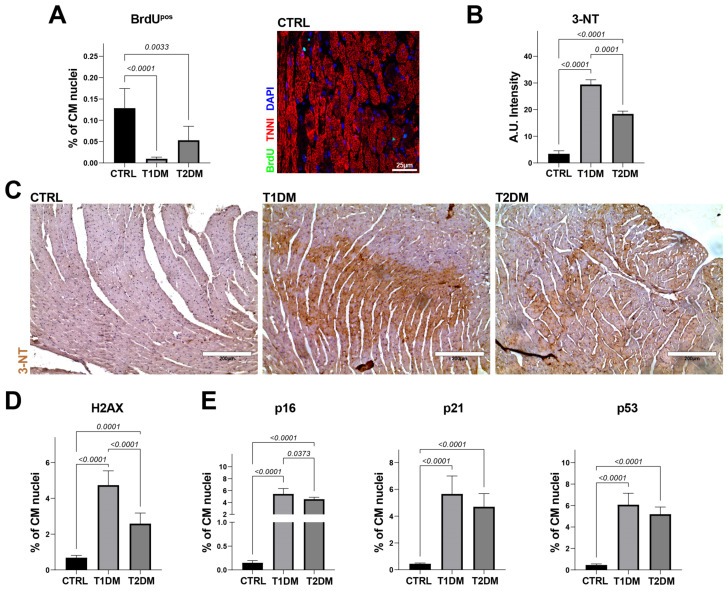
STZ-based T1DM and T2DM mouse models affected myocardial oxidative stress differently. (**A**) Bar graph and representative confocal image showing the percentages of BrdU-positive CMs in CTRL, T1DM and T2DM (CTRL, n = 7; T1DM, n = 8; T2DM, n = 9). Scale bar= 25 µm. (**B**) Bar graph showing quantification of 3-NT intensity levels in T1DM and T2DM cross-sections compared to CTRL tissue sections. (**C**) Representative light microscopy showing 3-NT-positive cardiomyocytes (3-NT, brown) from T1DM, T2DM and CTRL mice (CTRL, n = 7; T1DM, n = 8; T2DM, n = 9). Scale bar = 200 µm. (**D**,**E**) Bar graphs showing the percentages of γ-H2AX-, p16-, p21- and p53-positive CMs in cardiac cross-sections from CTRL, T1DM and T2DM (CTRL, n = 7; T1DM, n = 8; T2DM, n = 9). Data are mean ± SD.

To track myocardial cell regeneration, four weeks after STZ injection, the T1DM, T2DM and CTRL mice were implanted subcutaneously (between the two scapulae) with miniosmotic pumps to systemically release BrdU (Bromodeoxyuridine/5-bromo-2′-deoxyuridine, 50 mg/Kg/Day both) for 28 days. BrdU is an analogue of the nucleoside thymidine, whose cell incorporation in vivo is widely used to identify proliferating cells and, when administered continuously, as in this study, the detection of which provides the number of cumulative newly formed cells [27,28]. Cardiac sections from the T1DM and T2DM mice displayed a significantly lower percentage of BrdU-positive CMs when compared to the CTRL mice: 0.009 ± 0.004% vs. 0.05 ± 0.003% vs. 0.12 ± 0.02%, respectively (Figure 4A).

Overall, these data demonstrate that the conventional myocardial histopathological changes in the STZ-induced T1DM and T2DM mice involved high levels of myocardial cell hypertrophy, cell death and reactive fibrosis compared to those of the CTRL mice. Nevertheless, the T1DM mice had exaggerated pathological left ventricular remodeling, with more pronounced cell death and reactive hypertrophy and fibrosis when compared to the T2DM mice, as well as reduced myocardial cell regeneration. These findings suggest a more marked deleterious effect on left ventricular cell remodeling in STZ-based T1DM mouse models compared to those of STZ-based T2DM.

### 2.4. STZ-Based T1DM and T2DM Mouse Models Affected Oxidative Stress and Cell Senescence Differently

To assess whether different levels of oxidative stress and senescence underscore different effects of STZ-induced T1DM and T2DM mouse models on cardiac tissue remodeling and ventricular performance, we evaluated ROS production in the three groups of animals included in this study. Cardiac sections from the T1DM mice displayed a higher level of ROS, revealed with 3-NT immunostaining, compared to those of the T2DM and CTRL mice (Figure 4B,C). Moreover, the T1DM and T2DM mice significantly accumulated more DNA damage than did the CTRL mice, as demonstrated from the percentage of γ-H2AX-positive CM nuclei in the cardiac sections (Figure 4D). Interestingly, the percentage of γ-H2AX-positive CM nuclei was found to be ~twofold higher in the T1DM versus T2DM mice (Figure 4D).

**Figure 5:**
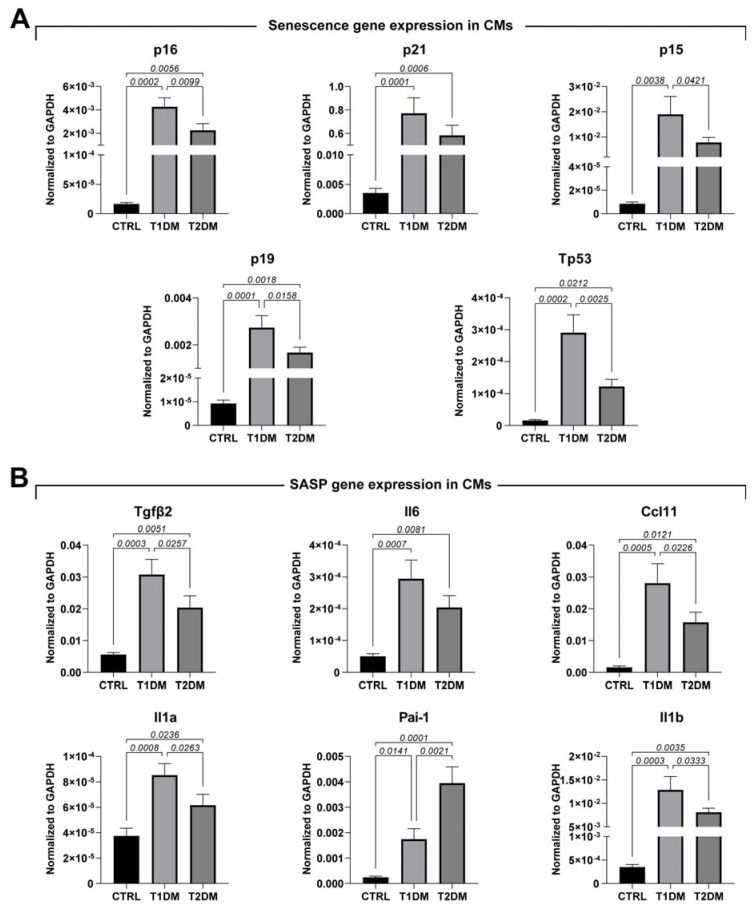
STZ-based T1DM and T2DM mouse models affected cardiac cell senescence differently. (**A**,**B**) Bar graphs showing the expressions of senescence and SASP markers in cardiomyocytes isolated from CTRL, T1DM and T2DM mice (n = 3). Data are mean ± SD.

Accordingly, heart sections from the T1DM and T2DM mice had an increased rate of CM-positive nuclei for classical biomarkers involved in cell-cycle inhibition and cell senescence, such as p16, p21 and p53, compared to the CTRL mice (Figure 4E). These results were further confirmed with RT-PCR on freshly isolated CMs from the T1DM, T2DM and CTRL mice, in which the expression levels of p16, p21, p15, p19 and p53 were evaluated and shown to be consistent with immunohistochemistry data (Figure 5A). Overall, these data demonstrated that T1DM mice develop higher levels of cellular senescence markers compared to T2DM mice (Figure 4D,E and Figure 5A).

To further investigate the involvement of cellular senescence, inducing chronic inflammation, in diabetes, we evaluated the senescence-associated secretory phenotype (SASP) [22,29,30] in freshly isolated CMs obtained from T1DM, T2DM and CTRL mice. The SASP has been postulated as a pathophysiological link between diabetes and senescence in cardiovascular diseases [22,23,31]. Thus, we evaluated the expression levels of Tgfβ2, IL-6, Ccl11, IL-1a and IL-1b, detecting higher levels of expression of these markers in T1DM compared to in T2DM. The only exception was PAI-1, as our results demonstrated a higher level of PAI-1 expression in the STZ-based T2DM mouse model when compared to the T1DM mice (Figure 5B).

Overall, these data suggest that DM determines a high level of oxidative stress in cardiac tissue, in both STZ-induced T1DM and T2DM mice, that is associated with DNA damage and cellular senescence. These phenomena are exaggerated in T1DM.

### 2.5. The Global Transcriptome Profile Showed Different Gene-Expression Signatures in the STZ-Based T1DM and T2DM Mouse Models

To assess whether STZ-induced T1DM and T2DM cardiomyopathy are characterized based on different patterns of gene expression, RNA extracted from cardiac sections from the T1DM and T2DM mice (n = 3 for each group) were processed for whole-heart transcriptome analysis through RNA sequencing. Cardiac sections from age- and sex-matched mice were used as the control (CTRL, n = 3). Once the libraries were obtained, the adapter-trimmed, high-quality reads aligned to the murine genome (mm10) data were processed to identify up- and downregulated gene sets, grouped with Gene Ontology resource tools, in different samples.

**Figure 6:**
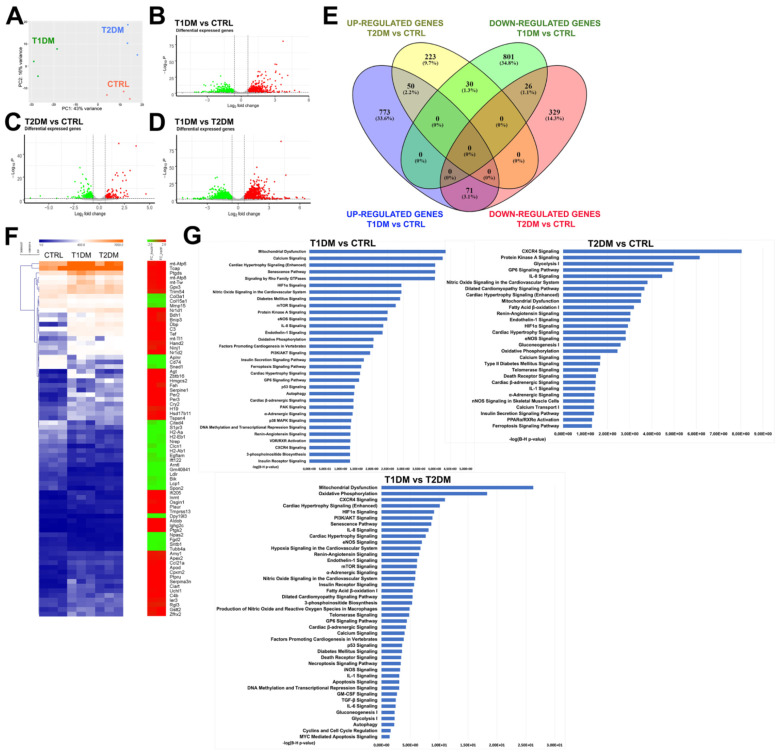
STZ-based T1DM and T2DM mouse models displayed different global transcriptome profiles and gene-expression signatures. (**A**) 2D-PCA analyses based on mRNA expression levels in CTRL, T1DM and T2DM samples (n = 3 per group). (**B**–**D**) Pairwise analysis of mRNA expressions between T1DM and CTRL, T2DM and CTRL and T1DM and T2DM, plotted in Volcano plots (|FC|1.5 and *p* ≤ 0.01). Red and green show the most significantly up- and downregulated mRNAs, respectively. (**E**) Venn diagram that shows the distributions of common, downregulated and upregulated genes in T1DM vs. CTRL, T2DM vs. CTRL and T1DM vs. T2DM. (**F**) Heat map showing similar fold changes of the common genes expressed in the two diabetic groups when compared to the same gene expressed in CTRL samples. (**G**) Functional categorization based on ingenuity pathway analysis (IPA) of the most significant canonical pathways generated in T1DM vs. CTRL, T2DM vs. CTRL and T1DM vs. T2DM. The ratio was calculated through division of the number of genes from our data set that mapped to each single pathway by the total number of genes included in the canonical pathway.

Principal component analysis (PCA) of global mRNA expression revealed the main axes of variance in the three cardiac samples and located the mRNA profiles of T1DM and T2DM at opposite poles (Figure 6A), showing at the same time a homogeneous clustering organization between the replicates of the two groups. A volcano plot enabled quick visual identification of genes, with large fold changes that were statistically significant. When a fold change of |FC| ≥ 1.5 was considered to be significant, the comparison of gene expression in the three samples revealed that 894 genes were upregulated in the T1DM versus CTRL mice (Figure 6B) and 857 were found to be downregulated in the same comparison (Figure 6B).

On the other hand, 303 upregulated and 426 downregulated genes were found in the T2DM samples when compared to the CTRL samples (Figure 6B). In comparison of the T1DM versus T2DM samples, the gene-expression analysis revealed 1920 upregulated and 2013 downregulated genes, indicating transcriptomic differences between the two DM animal models (Figure 6B). Distribution of the numbers of common, downregulated and upregulated genes in the T1DM versus CRTL and T2DM versus CRTL samples is shown in a Venn diagram (Figure 6C). We found 50 common upregulated genes and 26 common downregulated genes in the comparisons of the T1DM versus CRTL and T2DM versus CRTL samples (Figure 6C). The common gene expression between the two diabetic groups displayed similar fold changes when compared to the same gene expression in the CTRL samples (Figure 6C,D).

When the mRNA level expression was considered, the deregulated genes found in the T1DM versus CTRL mice were related to biological processes that were involved with mitochondrial dysfunction; calcium signaling; senescence pathways; and cardiac remodeling, hypertrophy, fibrosis, inflammation, oxidative stress and hypoxia (Figure 6E).

In the comparison between the T2DM and CTRL samples, the deregulated genes were involved in inflammation (CXCR4 signaling), cardiac contraction and relaxation (protein kinase A signaling, nitric oxide signaling and renin–angiotensin signaling), cardiac hypertrophy signaling and glucose metabolism signaling (glycolysis, gluconeogenesis and oxidative phosphorylation) (Figure 6E).

We also compared the deregulated genes in T1DM versus T2DM and found that they were related to biological processes involved with mitochondrial dysfunction, oxidative phosphorylation, inflammation, cardiac hypertrophy, cardiac fibrosis and senescence pathways (Figure 6E and Supplementary Figure S1).

**Figure 7:**
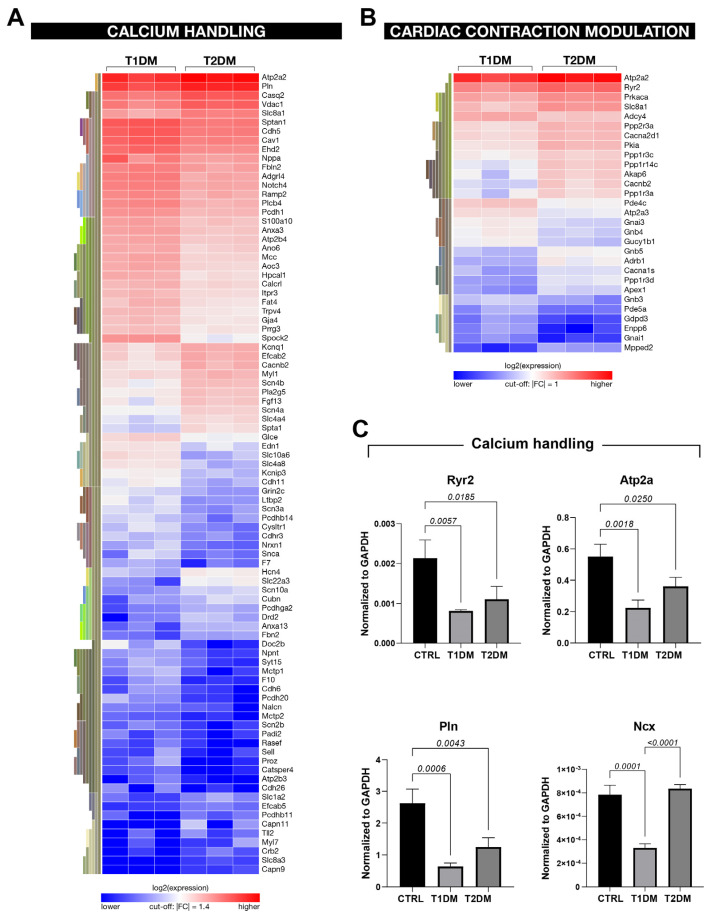
RNA-seq analysis from STZ-based T1DM and T2DM mouse models displayed modulation in the genes involved in calcium handling. (**A**,**B**) Heat maps showing differently expressed genes involved in the Ca2+ handling and cardiac contraction modulation processes in T1DM vs. T2DM. (**C**) Bar graphs showing the expressions of selected genes involved in Ca2+ handling in CMs isolated from CTRL, T1DM and T2DM mice (n = 3). Data are mean ± SD.

RNA-seq analysis revealed modulation in the genes involved in calcium handling in both T1DM and T2DM mice when compared to CTRL mice (Figure 6E). Remarkably, a number of genes involved with Ca^2+^ metabolism presented a more pronounced dysregulation in T1DM compared to T2DM (Figure 7A,B). Accordingly, the expression levels of the ryanodine receptor (Ryr2), SERCA2a (Atp2a), phospholamban (Pnl) and the Na^(+)^/Ca^(2+)^ exchanger (NCX) were significantly lower in T1DM mice compared to the type 2 counterpart, as assessed with RT-PCR in freshly isolated CMs from the T1DM, T2DM and CTRL mice (Figure 7C). These data demonstrate that the aforementioned genes were downregulated in the T1DM versus T2DM mice, confirming different modulations of calcium signaling in the two diabetic mouse models.

Overall, global transcriptome data demonstrated that the STZ-based T1DM and T2DM mouse models displayed different gene-expression signatures. The deregulated genes involved in the STZ-induced type 1 and type 2 diabetic mice were involved in mitochondrial dysfunction, glucose metabolism, pathological cardiac remodeling, inflammation and oxidative stress. Remarkably, the genes involved in the aforementioned pathways were found to be more severely deregulated in the T1DM mice compared to the T2DM mice.

## 3. Discussion

The main findings emanating from this study are that: i) Streptozotocin (STZ)-induced T1DM and T2DM affected left ventricular function and myocardial performance differently in mice, resulting in cardiomyopathy with heart failure with reduced ejection fraction for T1DM and with heart failure with preserved ejection fraction for T2DM; ii) Cardiomyocytes from the STZ-induced T1DM mice displayed exaggerated apoptotic death and reactive fibrosis and hypertrophy, along with increased cardiac oxidative stress and DNA damage, compared to those of the T2DM mice; iii) The STZ-induced T1DM mice also displayed a higher level of senescent cardiac cells and SASPs while showing severely reduced cardiomyocyte regeneration; iv) The STZ-based T1DM and T2DM mouse models differently affected the whole cardiac transcriptome, whereby several molecular pathways were commonly modulated, but at different levels, and other biological processes were distinctively activated.

DM is a chronic disease created via insufficient insulin production/secretion from the pancreas or via insulin resistance [32]. It is marked with uncontrolled hyperglycemia and accompanying metabolic derangements, eventually leading to severe damage to numerous tissue/organs, including the heart [32]. DM affects cardiac structure, tissue and function independently from other cardiovascular risk factors, causing, per se, a cardiomyopathy that leads to heart failure [8]. Diabetic cardiomyopathy is the focus of active basic and clinical medicine to understand the cellular and molecular basis of DM as well as to find effective therapeutic strategies [33]. Animal models of DM have been instrumental to understanding the pathogenesis and progression of this cardiomyopathy and extrapolating it to humans, but no ideal model exists, with several of them accounting for only specific features of the complexity that underlies DM cardiomyopathy [34,35]. DM in small animals may be developed through two principal mechanisms: use of specific drugs or genetic manipulation [36]. Streptozotocin has been widely used to create models of T1DM and T2DM [15,16], and these models have been generally used interchangeably to study DM cardiomyopathy, despite the fact that they have numerous clinical, immunological and genetic differences [11]. Therefore, we compared the anatomy, function, histology and whole transcriptomes of mouse hearts from STZ-induced DM models.

DM is associated with heart failure with both preserved ejection fraction (HFpEF) and reduced ejection fraction (HFrEFs) [37,38]. Here, we show that STZ-based T1DM and T2DM mouse models affected global left ventricular function and myocardial performance differently. The T2DM mice had normal ejection fraction but displayed diastolic dysfunction with significant increases in the E′ and E/E′ ratios. On the other hand, the T1DM mice had both diastolic and systolic dysfunction with reduced ejection fraction.

Cardiac remodeling, the key process that underlies heart failure, is classically defined based on ongoing cardiac death and reactive myocyte hypertrophy and interstitial fibrosis [39,40]. These cellular modifications are present in both the STZ-induced T1DM and T2DM mouse models. However, the levels of these pathological cellular events were significantly higher in the T1DM mice when compared to the T2DM mice, which may explain how T1DM-related cardiomyopathy reduces ejection fraction while the STZ-induced T2DM model causes prevalent diastolic cardiomyopathy.

The pathogenic effect of hyperglycemia in DM is classically mediated with an increased production of ROS that leads to tissue damage through activation of several stress-sensitive cellular pathways [39,40,41]. Experimental evidence has highlighted a direct link between oxidative stress and DM cardiomyopathy and has persuasively pointed to increased ROS production, which generates cardiac complications in diabetic patients [40,42]. Since the heart has low levels of free radical scavenging mechanisms, excessive formation of ROS results in induction of cardiovascular complications as central mechanisms for diabetes-associated inflammation and pathologic remodeling in the heart [39,40,43]. Defects in the antioxidant defense system further increase oxidative stress during the later stages of left ventricular dysfunction in DM cardiomyopathy [44,45]. Indeed, the hyperglycemic state leads to an increase in levels of oxidative-stress-induced DNA damage, leading to altered expressions of markers such as 3-Nitrotyrosine (3-NT), 8-hydroxy-2′-deoxyguanosine (8-OHdG) and γ-H2AX [22]. These markers have also been correlated to cardiac cellular senescence events in DM [22,46]. Cardiac oxidative stress and DNA damage were both found to be significantly higher in the mice with T1DM and T2DM when compared with euglycemic controls, and even higher in T1DM compared to T2DM. The latter was accompanied by a resultant pronounced cardiac cell senescence, leading to an exaggerated senescence-associated secretory phenotype (SASP), which could be key in the increased inflammatory state, as revealed from RNA-seq data of T1DM vs. T2DM. On the other hand, our data show that plasminogen activator inhibitor-1 (PAI-1) was specifically dysregulated in the SASP of T2DM. An emerging body of evidence has implicated plasminogen activator inhibitor-1 (PAI-1) in development of T2DM [33]. Studies in PAI-1 null-allele mice have highlighted better effects on insulin and glycemic measures when mice were fed a high-fat diet, as well as protective effects against development of obesity and insulin resistance [47,48]. Moreover, PAI-1 has been demonstrated to contribute to insulin resistance that in turn stimulates PAI-1 secretion from fat cells [49].

Accordingly, the RNA-seq analysis of the data of the whole cardiac transcriptome was in line with the anatomical, functional and histological data and documented that the T1DM and T2DM animal models displayed different expressions of genes involved in several biological processes and molecular pathways of glucose metabolism, inflammation, oxidative stress, the cell-death process and cardiac contraction and hypertrophy. Furthermore, the RNA-seq bioinformatics analysis highlighted that DM severely affects the biological pathways involved with Ca^2+^ handling. Abnormality of the latter, resulting from DM-induced cardiac renin–angiotensin system (RAS) activation, is involved in the pathogenesis of LV dysfunction [50]. LV relaxation and contraction are mediated with cytosolic Ca^2+^ handling and the sarcoplasmic reticulum through the involvement of modulation of key genes, including Ryr2, Atp2a, Pnl and NCX [50,51], which were all significantly modulated via STZ-induced DM, in the T1DM model in particular. Furthermore, RNA-seq analysis pointed at significant differences in the mTOR and autophagy pathways in cardiomyopathy from the T1DM versus T2DM models. Numerous studies that also employed STZ-derived DM models have demonstrated that autophagy, an intracellular system for protein degradation that depends on mTOR signaling, is impaired in the DM heart, suggesting that autophagy is a potential target to reduce cardiac maladaptive alterations in patients with DM [52,53].

In conclusion, the present study offers a head-to-head comparison of the two classical models of STZ-induced DM in mice, providing a cellular, molecular and functional fingerprint of the relative cardiomyopathy. These differences should be taken in account when choosing an animal model of DM to study diabetic cardiomyopathy, and could be of key relevance when addressing the bases of and potential therapies for specific features that underlie diabetic heart disease.

## 4. Materials and Methods

### 4.1. Animals

All experimental procedures on mice were approved by the Magna Graecia Institutional Review Boards on Animal Use and Welfare (authorization number 368/2016-PR, released on 8 April 2016, extended on 25 November 2021) and performed according to the Guide for the Care and Use of Laboratory Animals from directive 2010/63/EU of the European Parliament. The mice were housed under controlled conditions of 25 °C, 50% relative humidity and a 12 h light (6:00–18:00) and 12 h dark cycle, with water and food (containing 18.5% protein) available ad libitum. All mice received human care and all efforts were made to minimize animal suffering. Before any invasive procedure, the mice were anesthetized with i.p. injections of tiletamine/zolazepam (80 mg/kg) or inhaled isoflurane (isoflurane, 1.5%; oxygen, 98.5%; Iso-Vet Piramal Healthcare, Aurora, ON, Canada).

To induce T1DM (n = 15), 10-week-old C57BL/6J male (n = 8) and female (n = 7) mice (Charles River) were treated with a single high dose of streptozotocin (STZ, 200 mg/kg i.p.). The powder was dissolved in 0.05 M of citrate buffer, pH 4 [22].

To induce T2DM (n = 15), 6-week-old C57BL/6J male (n = 8) and female (n = 7) mice (Charles River, Wilmington, DE, USA) were fed with a 60 kcal% high-fat diet (HFD) for 3 weeks. During the fourth week, the mice received 4 consecutive daily injections of low-dose streptozotocin (STZ, 40 mg/kg i.p.) [22].

Sex- and age-matched C57BL/6J mice (n = 20) were used as controls (CTRL: normal-chow-diet mice, n = 10; high-fat-diet mice, n = 10). Four mice in the T1DM group (~30%) and three mice in the T2DM group (20%) died during the first week after STZ injection.

Three animals from each group were used for adult cardiomyocyte isolation. All of the other mice were used for immunohistochemistry and RNA-seq analysis (see below).

The mice were subcutaneously implanted with miniosmotic pumps (ALZET, Cupertino, CA, USA) to systemically release BrdU (50 mg/Kg/day) prepared through dissolution of BrdU powder in 50% deionized water and 50% DMSO.

Eight weeks after the STZ injections, all animals were sacrificed, and the hearts were processed either for immunohistochemistry analysis and RNA isolation or for cardiomyocyte (CM) isolation [23].

### 4.2. Mouse Cardiomyocyte Isolation

CMs were isolated as established and reproduced in our laboratory through standard enzymatic dissociation from the hearts of each group of mice (CTRL, T1DM and T2DM mice), as previously described [28,54,55].

### 4.3. Echocardiography

Mice that underwent echocardiographic evaluation were prepared as previously described in detail [22,23,27]. All echo images and videos were obtained from the mice at heart rates > 400 b.p.m. Echocardiographic images and videos were obtained with a Vevo 3100 system (Visualsonics, Inc., Toronto, Canada) equipped with a MX550D ultra-high-frequency linear-array transducer (22–55 MHz) [22,23,27]. B-mode, M-mode and speckle-tracking images were analyzed through Vevo LAB analysis software Version 3.2.0 (VisualSonics, Amsterdam, The Netherlands) as previously described [23,27]. The n-value for each experimental group is specified in the figure legends.

### 4.4. Tissue Harvesting, Histology and Immunohistochemistry

For immunohistochemistry analysis, the abdominal aorta was cannulated and the heart arrested in diastole using a cadmium chloride/potassium solution. Mouse tissue specimens were fixed and embedded in an optimal cutting temperature (OCT) compound for immunohistochemical analysis.

Tissues were cut into 5 µm cross-sections, respectively, and processed for both fluorescent as well as chromogenic immunohistochemistry according to specific analysis.

For bioquantification of fibrosis, OCT sections were stained with Picrosirius red. Staining was performed as per the manufacturer’s instructions (Bioptica, Milan, Italy). Hematoxylin and eosin (H&E, Bioptica) were used to evaluate the cellular architectures of the samples.

For detection of p16-, γ-H2AX-, 3-NT-, p53- and p21-positive CMs, immunohistochemical analysis was performed using anti-p16 (1:100 dilution; Santa Cruz, Dallas, TX, USA), anti-γ-H2AX (1:200 dilution; Cell Signaling, Danvers, MA, USA), 3-NT (1:100; Millipore, Taufkirchen, Germany), anti-p53 (1:100 dilution; Santa Cruz, Dallas, TX, USA) and anti-p21 (1:100 dilution; Santa Cruz) antibodies. Positive reactions were visualized using a labeled polymer–HRP complex and a 3,3′-diaminobenzidine tetrahydrochloride (DAB) chromogen (EnVision + Dual Link System-HRP, DAKO, Santa Clara, CA, USA). Sections were then counterstained with hematoxylin and examined with light microscopy (LEICA, Wetzlar, Germany, DMI3000B). The numbers of p16-, p21-, p53- and γ-H2AX-positive CMs were expressed as percent fractions of the total CM nuclei.

For immunofluorescent staining, antigen retrieval was achieved using a target retrieval solution with a citrate pH of 6 (DAKO). The following primary antibodies were used: anti-BrdU (1:50 dilution; Sigma-Aldrich, Taufkirchen, Germany), anticardiac troponin I (1:100 dilution; Abcam, Cambridge, UK). Each primary antibody was revealed with respective antimouse IgG or antirabbit IgG (1:100 dilution; Jackson Immunoresearch, Ely, UK). The nuclei were counterstained with DAPI (4,6-diamidino-2-phenylindole; Sigma) DNA binding dye at 1 µg/mL.

An In Situ Cell Death Detection Kit (TdT, Sigma-Aldrich, ST. Luis, MO, USA) was used as per the manufacturer’s instructions for detection of apoptosis-positive CM nuclei. BrdU and TdT fluorescence quantifications were obtained through manual counting of respective histological samples, and the numbers of BrdUpos and TdTpos CMs were, respectively, expressed as percent fractions of the total CM nuclei [23,27].

CM cross-sectional area was measured through immunofluorescence staining for wheat germ agglutinin (WGA) of the Alexa Fluor 647 conjugate (1:200 dilution; Invitrogen, Waltham, MA, USA) and digital analysis of acquired cardiac cross-section images. CM diameter was measured across the nucleus on three transverse sections (~500 myocytes/animal were sampled), as previously described [27]. All immunofluorescence staining was acquired and analyzed using confocal microscopy (LEICA, Wetzlar, Germany, TCS SP5 and SP8).

### 4.5. Quantitative RT-PCR (qPCR)

RNA was extracted from CMs using the TRIzol Reagent (Ambion, Waltham, Ma, USA) and quantified using a Nanodrop 2000 Spectrophotometer (Thermo Fisher Scientific, Waltham, MA, USA). Reverse transcription was performed with 0.5–1 µg of RNA, using the HighCapacity cDNA Kit (Applied Biosystems, Waltham, MA, USA). Quantitative qPCR was performed using TaqMan Primer or Probe sets (Applied Biosystems or Eurofins Genomics, Ebersberg, Germany) (see Table 2) using the StepOne Plus Real-Time PCR System (Applied Biosystems, Waltham, MA, USA). All reactions were carried out in triplicate.

### 4.6. RNA Sequencing

#### 4.6.1. RNA Extraction

RNA was extracted from whole heart sections of the CTRL, T1DM and T2DM mice, starting from OCT-embedded tissues. Ten sections, each of 20 µm in thickness, were collected in 1.5 mL tubes, and RNA extraction was performed using an RNA Purification Kit (Norgen, Thorold, ON, Canada).

#### 4.6.2. Library Preparation

Libraries were generated using depleted RNA obtained from 1 μg of total RNA with a TruSeq Sample Preparation RNA Kit (Illumina, Inc., San Diego, CA, USA), according to the manufacturer’s protocol without further modifications, as previously described [56,57].

#### 4.6.3. Sequencing

All libraries were sequenced on the Illumina HiSeq 1000, generating 100 bp paired-end reads. The libraries were divided into two groups depending on how they were prepared.

### 4.7. RNA-Seq Data Analysis

All FastQ files were quality checked using FastQC software (v0.11.9) [58]; then, adapter sequences were removed and low-quality reads were filtered out using Cutadapt software (version 1.18) [56] with parameters set as follows: quality cutoff, 20; minimum length, 20. The resulting high-quality reads were then mapped to the mouse reference genome (GRCm39). This alignment was performed using default parameters with STAR software (version 2.7.10b) [57]. Then, the number of reads that mapped to each transcript within the reference was computed with FeatureCounts (v2.0.1) [59]. The counts were then imported in in R package DESeq2 (v1.38.1) (R version 3.6.3) [60], and differential gene-expression analysis was performed through comparison of each experiment condition with the controls. Differential expression was reported as |fold change| ≥ 1.5 along with associated adjusted *p* values (FDR ≤ 0.05), computed according to Benjamini–Hochberg [61] as described in Salvati et al., 2019 [62]. For Gene Ontology (GO) analysis of DE genes, Ingenuity Pathway Analysis Software (IPA 84978992, Ingenuity^®^ Systems, www.ingenuity.com, accessed on 20 December 2022) was used, and only functions and pathways that showed −log(B-H *p*-value) ≥ 1.3 were considered.

### 4.8. Statistical Analysis

Data are reported as mean ± SD. Significance between any 2 groups was determined with Student’s *t*-test and in multiple comparisons with analysis of variance (ANOVA), using GraphPad Prism version 9.4.0 for Windows (GraphPad Software Version 9.4.0, San Diego, CA, USA). In the event that ANOVA justified posthoc comparisons between group means, these were corrected using the Tukey multiple comparison test. Differences of *p* < 0.05 were considered statistically significant.

## Figures and Tables

**Table 1:** Echocardiographic data.

	CTRL (n = 10)	T1DM (n = 11)	T2DM (n = 12)	*p*-Value
HR (bpm)	446.67 ± 24.16	463.55 ± 49.13	442.25 ± 40.34	0.428
LVEDD (mm)	4.06 ± 0.08	4.40 ± 0.20	3.95 ± 0.23	<0.001
LVESD (mm)	2.82 ± 0.07	3.41 ± 0.19	2.75 ± 0.30	<0.001
EF (%)	58.33 ± 2.32	45.63 ± 3.84	58.41 ± 6.55	<0.001
FS (%)	30.45 ± 1.63	22.60 ± 2.29	30.57 ± 4.49	<0.001
GLS (%)	−21.49 ± 1.33	−13.93 ± 3.38	−18.49 ± 2.99	<0.001
E (mm/sec)	639.98 ± 88.09	702.06 ± 78.32	587.87 ± 73.18	0.008
A (mm/sec)	399.20 ± 54.74	422.69 ± 57.17	411.68 ± 80.27	0.756
E/A	1.62 ± 0.3	1.65 ± 0.19	1.47 ± 0.27	0.256
E′ (mm/sec)	−25.18 ± 3.10	−21.68 ± 3.97	−18.44 ± 4.96	0.005
E/E′	25.6 ± 3.5	33.10 ± 5.62	33.35 ± 7.24	0.009
E′/A′	1.15 ± 0.26	0.77 ± 0.11	0.97 ± 0.23	0.002
IVSd (mm)	0.65 ± 0.06	0.62 ± 0.05	0.59 ± 0.04	0.067
LVPWd (mm)	0.63 ± 0.07	0.64 ± 0.07	0.62 ± 0.05	0.628

Values are mean ± SD. HR, heart rate; LVEDD, left ventricular end diastolic diameter; LVESD, left ventricular end systolic diameter; EF, ejection fraction; FS, fractional shortening; GLS, global longitudinal strain; IVSd, interventricular septum at end diastole; LVPWd, left ventricular posterior wall at end diastole.

**Table 2:** List of primers.

Gene	Sequence (5′ -> 3′)
mGapdh	Fwd-CTCCACTCTTCCACCTTCG-
	Rev-GCCTCTCTTGCTCAGTGTCC-
mTgfb2	Fwd-CCGCATCTCCTGCTAATGTTG-
	Rev-AATAGGCGGCATCCAAAGC-
mNppa	Fwd-CTGATGGATTTCAAGAACCTGCT-
	Rev-TCTCAGAGGTGGGTTGACCT-
mp21	Fwd-AACATCTCAGGGCCGAAA-
	Rev-TGCGCTTGGAGTGATAGAAA-
mp16	Fwd-GTGTGCATGACGTGCGGG-
	Rev-GCAGTTCGAATCTGCACCGTAG-
mp15	Fwd-AGATCCCAACGCCCTGAAC-
	Rev-CCCATCATCATCACCTGGATT-
mp19	Fwd-GCTCTGGCTTTCGTGAACATG-
	Rev-TCGAATCTGCACCGTAGTTGAG-
mMybpc2	Fwd-CTGCTAGGGCCTGGTTAGAG-
	Rev-CCTTTTTGGCCGCTGGTTTA-
mIl-6	Fwd-TGAGAAAAGAGTTGTGCAATGG-
	Rev-GGTACTCCAGAAGACCAGAGG-
mCcl11	Fwd-TGCAGAGCTCCACAGCGCTT
	Rev-GGGTGAGCCAGCACCTGGGA
mPai-1	Fwd-GGCCATTACTACGACATCCTG
	Rev-GGTCATGTTGCCTTTCCAGT
mIl1b	Fwd-TGCCACCTTTTGACAGTGATG
	Rev-TGATGTGCTGCTGCGAGATT
mGja1	Fwd GGT GAT GAA CAG TCT GCC TTT CG
	Rev GTG AGC CAA GTA CAG GAG TGT G
mCol1a1	Fwd CCT CAG GGT ATT GCT GGA CAA C
	Rev CAG AAG GAC CTT GTT TGC CAG G
mCol1a2	Fwd TTC TGT GGG TCC TGC TGG GAA A
	Rev TTG TCA CCT CGG ATG CCT TGA G
mCol3a1	Fwd GAC CAA AAG GTG ATG CTG GAC AG
	Rev CAA GAC CTC GTG CTC CAG TTA G
mMyl7	Fwd AGG AAG CCA TCC TGA GTG CCT T
	Rev CAT GGG TGT CAG CGC AAA CAG T
mCasp3	Fwd GGA GTC TGA CTG GAA AGC CGA A
	Rev CTT CTG GCA AGC CAT CTC CTC A
mBcl2	Fwd CCT GTG GAT GAC TGA GTA CCT G
	Rev AGC CAG GAG AAA TCA AAC AGA GG
mBax	Fwd AGG ATG CGT CCA CCA AGA AGC T
	Rev TCC GTG TCC ACG TCA GCA ATC A
mFoxo3	Fwd CCT ACT TCA AGG ATA AGG GCG AC
	Rev GCC TTC ATT CTG AAC GCG CAT G
mFoxo1	Fwd CTA CGA GTG GAT GGT GAA GAG C
	Rev CCA GTT CCT TCA TTC TGC ACT CG
mIL1a	Fwd AGGGAGTCAACTCATTGGCG
	Rev TGGCAGAACTGTAGTCTTCGT
mTp53	Fwd ATGGCCATCTACAAGAAGTCACAG
	Rev ATCGGAGCAGCGCTCATG
mMyh7	Fwd GCTGGAAGATGAGTGCTCAGAG
	Rev TCCAAACCAGCCATCTCCTCTG
mRyr2	Fwd ACCTACTCCGAAGGCTGGTGTT
	Rev TTCTTCCGAGGCAGCACCAAAG
mAtp2a	Fwd GTGAAGTGCCATCAGTATGACGG
	Rev GTGAGAGCAGTCTCGGTAGCTT
mPnl	Fwd GGACCAAAGGAACTTGCCAGCT
	Rev CAACAGGCAGCCAAATGTGAGC

## Data Availability

All data are available within this article except for RNA Seq complete dataset that are publicly available at Arrayexpress (https://www.ebi.ac.uk/biostudies/arrayexpress) with the accession number: E-MTAB-12558.

## Supplementary Materials

The following supporting information can be downloaded at: https://www.mdpi.com/article/10.3390/ijms24021132/s1.
