# Supplementary material for: Streptozotocin-Induced Type 1 and 2 Diabetes Mellitus Mouse Models Show Different Functional, Cellular and Molecular Patterns of Diabetic Cardiomyopathy
Source: Int J Mol Sci. 2023 Jan 6;24(2):1132. doi: 10.3390/ijms24021132 (PMC9860590; doi:10.3390/ijms24021132)
Supplement: Supplementary file 1 [file ijms-24-01132-s001.zip › Supplementary new_R2-corrected.pdf]

International Journal of *Molecular Sciences*

MDPI

Supplementary

Streptozotocin-induced Type 1 and 2 Diabetes Mellitus Mouse Models Show Different Functional, Cellular and Molecular Patterns of Diabetic Cardiomyopathy

Fabiola Marino[1,†], Nadia Salerno[2,†], Mariangela Scalise[1], Luca Salerno[1], Annalaura Torella[3], Claudia Molinaro[2], Antonio Chiefalo[1], Andrea Filardo[2], Chiara Siracusa[2], Giuseppe Panuccio[2], Carlo Ferravante[4], Giorgio Giurato[4], Francesca Rizzo[4], Michele Torella[5], Maria Donniacuo[6], Antonella De Angelis[6], Giuseppe Viglietto[1], Konrad Urbanek[7], Alessandro Weisz[4], Daniele Torella[1,*] and Eleonora Cianflone[2,*]

1 Department of Experimental and Clinical Medicine , Magna Graecia University, 88100 Catanzaro, Italy

2 Department of Medical and Surgical Sciences, Magna Graecia University, 88100 Catanzaro, Italy

3 Department of Precision Medicine, University of Campania "Luigi Vanvitelli", 80138 Naples, Italy

4 Department of Medicine, Surgery and Dentistry 'Scuola Medica Salernitana', University of Salerno, 84081 Salerno, Italy

5 Department of Translational Medical Science, University of Campania "L. Vanvitelli", 80138 Naples, Italy

6 Department of Experimental Medicine, University of Campania "L. Vanvitelli", 80138 Naples, Italy

7 Department of Molecular Medicine and Medical Biotechnology, Federico II University, 88121, Naples, Italy

* Correspondence: dtorella@unicz.it (D.T.); cianflone@unicz.it (E.C.); Tel.: +39-0961369-7564 (ext. 4185)
† These authors contributed equally to this work.

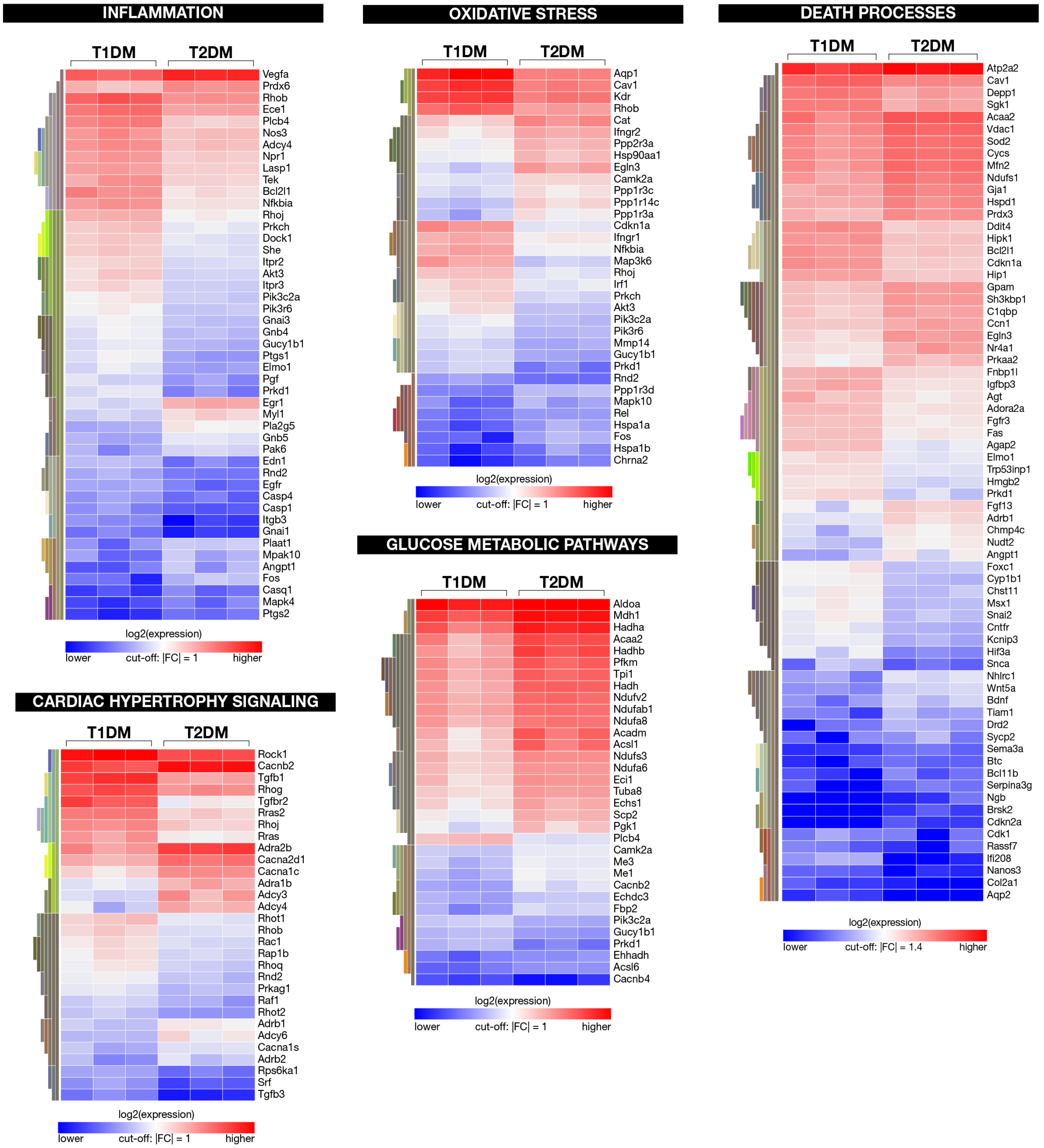

Supplementary Figure S1. RNA-seq analysis from STZ-based DMT1 and DMT2 mouse models displays a modulation in genes involved in selected biological processes. Heatmaps showing differentially expressed genes involved in inflammation, oxidative stress, death processes (Apoptosis, Autophagy and Necrosis), cardiac hypertrophy signaling and glucose metabolic pathways in T1DM vs. T2DM.
